# Influence of Interface Modification on the Moisture Absorption and Thermal Resistance of Ramie Fiber/Degradable Epoxy Composites

**DOI:** 10.3390/ma17081779

**Published:** 2024-04-12

**Authors:** Jingqi Geng, Yingchun Cai

**Affiliations:** Key Laboratory of Bio-Based Material Science and Technology of Ministry of Education, School of Material Science and Engineering, Northeast Forestry University, Harbin 150040, China; gengjingqi@nefu.edu.cn

**Keywords:** natural fiber, degradable epoxy, interfacial strength, thermal resistance

## Abstract

Natural fiber/degradable epoxy composites have received much attention for their advantages of low carbon emissions, low environmental pollution, and utilization of renewable resources. However, the poor interfacial bonding strength and inferior moisture resistance of natural fiber/degradable epoxy composites restrict their application areas. In order to improve the moisture and heat resistance of natural fiber/degradable epoxy resin-based composites, this study modified the surfaces of ramie fibers with hydroxylated carbon nanotubes, silane coupling agents, and sodium hydroxide, respectively. Three types of modified ramie fiber/degradable epoxy composites, namely F-CN-DEP, F-Si-DEP, and F-OH-DEP, were prepared using a winding forming process. The water absorption rate and short-beam shear strength of the materials were tested under different environments, and the fiber morphology and thermal–mechanical properties of the materials were investigated by scanning electron microscopy (SEM) and dynamic mechanical analysis (DMA). The results show that F-CN-DEP exhibited the lowest moisture absorption rate; the highest shear strength, of 43.8 MPa; and a glass transition temperature (*T*_g_) of 121.7 °C. The results demonstrate that carbon nanotubes on the fiber surface can improve the interfacial stability of ramie fiber/degradable epoxy composites in humid and hot environments. These results give guidelines for the development of natural fiber/degradable epoxy composites.

## 1. Introduction

Natural fiber/degradable epoxy composites have advantages such as low carbon emissions, low environmental pollution, and the utilization of renewable resources [1,2,3]. Conducting research on their applications in areas such as construction and transportation is of great social significance and economic value in promoting sustainable development and the development of green materials [4]. However, the molecular structure of degradable epoxy resin contains a large number of ester and/or ether bonds, which are prone to being adsorbed by water molecules and forming hydrogen bonds [1,5,6]. As a resin matrix, it has relatively high moisture absorption, which is detrimental to the stability of composite materials in humid and hot environments.

The hydroxyl and carboxyl groups on the surfaces of natural fibers easily form hydrogen bonds with water molecules, and the abundant porous structures inside the fibers facilitate water adsorption [7,8,9]. These issues result in poor interfacial bonding strength and inferior moisture resistance of natural fiber/degradable epoxy composites [3], severely limiting their application range and performance. Studies of the literature have shown that due to the hydroxyl groups on the surfaces of natural fibers and the water absorption of degradable epoxy resin [10,11], interfacial bonding strength is weak, leading to a decrease in the mechanical properties and durability of the composite materials. Moreover, the dimensional stability of the composite materials is poor in humid and hot environments [12,13]. Water absorption first increases the hydrophilicity of the fiber surface, affecting the interaction between the fiber and the resin. Secondly, the expansion of the resin matrix after water absorption affects the interface morphology between the fiber and the resin, resulting in shear and interface delamination, thereby reducing the interfacial bonding strength. In addition, the expansion of the resin matrix after water absorption may increase the gaps between the fiber and the resin, further promoting the penetration of water molecules into the composite material. Furthermore, the expansion of the resin matrix after water absorption also leads to the concentration of tensile stress at the interface between the fiber and the resin, resulting in interface fracture and fiber debonding.

Fortunately, related researchers have proposed different treatment methods to enhance the interface force between fibers and epoxy. Huner [14] examined the impacts of various chemical surface treatments (alkali, acetic anhydride, and silane) on flax fiber-reinforced epoxy composites. That study revealed that alkali-treated, flax fiber-reinforced epoxy composites exhibited the highest strength values in both tensile and flexural tests. Furthermore, treatments involving acetic anhydride and silane induced minor alterations in flexural properties. Milosevic et al. [15] explored the tribological properties of an epoxy composite material reinforced with abaca fibers, examining the influence of fiber orientation on the composite’s tribological performance. That study investigated an epoxy resin with exceptionally low viscosity reinforced by long abaca fibers treated with NaOH under various operational conditions. Composite specimens were created with unidirectional abaca fibers reinforcing the epoxy resin, resulting in fibers oriented in three directions, parallel (P-O), anti-parallel (AP-O), and normal (N-O), while maintaining the sliding direction constant. However, the issue of poor bonding between degradable epoxy and natural fibers needs to be understood and solved before the application of fiber/degradable epoxy composites.

In the present work, ramie fibers reinforced by degradable epoxy resin were prepared using a fiber winding process. The surfaces of these fibers were modified with different chemical treatments, including sodium hydroxide, a silane coupling agent, and hydroxylated carbon nanotubes. The water absorption rate and short-beam shear strength of the materials were tested under various conditions, while the fiber morphology and thermal–mechanical properties were observed utilizing SEM and DMA. The findings revealed that F-CN-DEP exhibited the lowest moisture absorption rate; the highest shear strength, of 43.8 MPa; and a glass transition temperature (*T*_g_) of 121.7 °C. These results indicate that the presence of carbon nanotubes on fiber surfaces can enhance the interfacial stability of ramie fiber/degradable epoxy composites in humid and high-temperature environments. These outcomes provide guidance for the advancement of natural fiber/degradable epoxy composites.

## 2. Materials and Methods

### 2.1. Materials

Degradable epoxy resin was provided by Aida Suo Wuhu Co., Ltd., Wuhu, China. Ramie fiber was obtained from Qichun County Dongshen Textile Raw Materials Co., Ltd., Huanggang, China. A silane coupling agent (KH-560) was supplied by Hangzhou Jessica Chemical Co., Ltd., Hangzhou, China. Hydroxylated carbon nanotubes were purchased from Beijing Deke Island Gold Technology Co., Ltd., Beijing, China. Sodium hydroxide (NaOH, analytical grade) was purchased from Changshu Yangyuan Chemical, Changshu, China.

### 2.2. Sample Preparation

First, the ramie fibers were soaked in a solution of sodium hydroxide (1 mol/L), KH-560, and hydroxylated carbon nanotubes for 45 min, respectively. After soaking, the fibers were air-dried and then dried in a blast oven at 80 °C for 12 h. Next, the modified ramie fibers were used to make unidirectional boards with degradable epoxy resin using a fiber winding process. Natural fibers with different treatment methods were added to each grade at 50% of the total volume of the composite. In addition, the abbreviations of the composite materials obtained by different processing methods are shown in Table 1.

### 2.3. Water Absorption Test

Samples with dimensions of 100 mm × 10 mm × 2 mm were prepared to determine the water absorption according to the ASTM D570-98 method [16,17]. All samples underwent initial drying at 105 °C for 1 h in an oven, and the weight obtained during this process was recorded as the dry weight (*W_d_*) of the specimens. Subsequently, the specimens were immersed in a 3.5% NaCl solution at both room temperature and 60 °C. The weights of the specimens immersed in the 3.5% NaCl solution were regularly measured using a Sartorius weighing scale with a precision of 1 mg. The percentage weight gain at any given time, *M_t_*, resulting from water absorption was calculated using Equation (1), as follows:

(1) $$M_{t}\left(\%\right)=\frac{\left(W_{w}-W_{d}\right)}{W_{d}}\times 100$$

Here, *W_w_* represents the weight of the wet material. The equilibrium moisture absorption or maximum moisture content, *M_m_*, was determined as the average value of multiple consecutive measurements, considering the maximum values obtained after exposure for a specific period.

The increase in weight due to water absorption, denoted as *M_t_*, can alternatively be represented by two parameters: the diffusion coefficient or diffusivity, *D*, and the maximum water content, *M_m_*, as expressed in the following, Equation (2) [18]:

(2) $$M_{t}=M_{m}\left\{1-\frac{8}{\pi^{2}}exp\left[\left(\frac{Dt}{h^{2}}\right)\pi^{2}\right]\right\}$$

In this equation, *t* represents time and *h* stands for the thickness of the sample. The diffusion coefficient, *D*, can be determined from the initial linear segment of the water absorption curve, which corresponds to the slope of *M_t_* versus *t*^1/2. Furthermore, to assess the degrees of recovery in the flexural and impact properties of the specimens, an additional step was conducted by re-drying the specimens in an oven at 105 °C for 1 h.

### 2.4. Mechanical Properties

The winding tension was set at 20 N, and the curing process was carried out at 100 °C for 2 h and 150 °C for 1 h. Finally, the samples were machined using a CNC machine. The fiber volume fraction was calculated to be 52%. Tensile tests were performed following the ASTMD 638-02 standard [19] to ascertain the tensile strengths of the WFP composites [20]. Standard specimens were sized at 165 × 13 × 3 mm. The tests were conducted at a crosshead speed of 10 mm/min, with results obtained from three specimens to calculate the average value. Additionally, an impact test (using a 10 kN load cell) adhered to the ASTM D5942-96 standard [21,22], employing a Charpy impact machine with un-notched specimens. The dimensions of the impact specimens were 75 × 10 × 3 mm. In each instance, five samples underwent testing and the average values were reported.

Dynamic mechanical analyses (DMAs) were performed using a TA DMA Q800 apparatus (New Castle, DE, USA). Specimens measuring (30 ± 0.2) mm × (5 ± 0.2) mm × (0.5 ± 0.05) mm were securely mounted on a film tension clamp and subjected to testing in “multifrequency strain” mode, ranging from 25 to 425 °C with a heating rate of 3 °C min^−1^, at a frequency of 1.0 Hz.

Stress–strain curves were obtained with a Labthink C610 auto tensile tester at room temperature with a stretch rate of 10 mm min^−1^. All composites were around 100 mm × 15 mm × 1.6 mm. At least five specimens were tested to obtain the average value for each sample.

### 2.5. Scanning Electron Microscope

The surfaces of the different as-prepared composites were observed by using scanning electron microscopy (SEM; SU8010, Hitachi, Tokyo, Japan) with an acceleration voltage of 12 kV. The as-prepared composites were glued into the substrate with carbon glue. Then, the surface of each specimen was covered with a thin layer of gold–palladium through sputter coating in a vacuum chamber to enhance conductivity prior to examination.

### 2.6. Thermal Properties

The thermal stability of the as-prepared composites was assessed through thermogravimetric analysis (TGA) using a Pyris 1 TGA Perkin Elmer Model apparatus, Waltham MA, USA. Approximately 5 mg of the sample, sealed in an aluminum pan, underwent heating from room temperature to 550 °C at a rate of 10 °C/min under a nitrogen atmosphere. The onset temperature of degradation and the residual mass were determined from the TGA curve.

## 3. Results

### 3.1. Water Absorption Behavior

**Figure 1:**
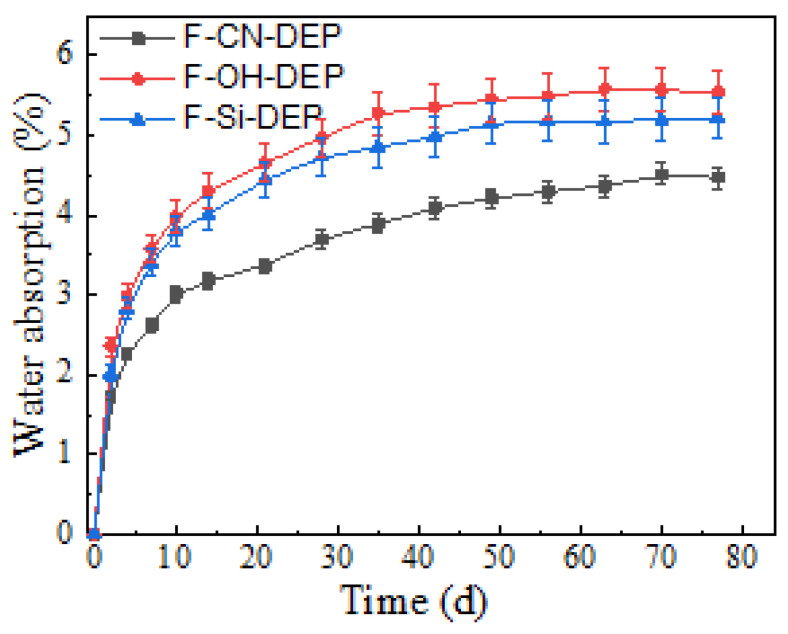
The moisture absorption rates of the different composites at room temperatures. The error bars represent the standard deviations of five representative measurements.

Figure 1 shows the variation in the percentages of water absorption (*M_t_*) of the three composite material samples as a function of time in a 3.5% NaCl solution at room temperature. All the water absorption curves can be divided into three stages. During the first stage (0–14 days), the weight of the samples increased linearly due to the water absorption. This was the penetration stage, during which water molecules rapidly entered the material through the pore structure between the composite material resin matrix and the fiber–resin interface. In the second stage (14–42 days), the water absorption rate slowed down until the third stage, where the water absorption weight reached a stable state. At that point, water molecules had filled the pores inside the material and diffused along the concentration gradient through the interface between the fibers and the matrix and the gaps between the fibers. This demonstrated that the water absorption behavior of these three composites obeys Fick’s law [23]. The maximum water content, *M*_m_, and values of diffusivity are summarized in Table S1.

Ramie fibers contain some oxygen-containing hydrophilic functional groups on their surface, such as hydroxyl (-OH), aldehyde (-CHO), and ketone (-C=O) groups, which can form hydrogen bonds with water molecules and accelerate them into the fiber. Additionally, ramie fibers have a porous structure that can adsorb and store water molecules. Therefore, compared to glass (carbon) fiber/epoxy resin-based composite materials, the moisture absorption rate of ramie fiber/degradable epoxy-based composite materials is significantly higher. In comparing the three curves, it can be seen that the moisture absorption rate and amount of the F-CN-DEP composite material are significantly lower than those of the F-Si-DEP and F-OH-DEP composite materials. The F-Si-DEP and F-OH-DEP reached saturation after 42 days of water absorption, whereas the F-CN-DEP was still slowly absorbing water. The main reasons for this are as follows: First, carbon nanotubes have a high specific surface area, which can adsorb a large number of water molecules and slow down the penetration rate of water molecules in the composite material, thereby reducing the chance of water molecules entering the composite material. Second, carbon nanotubes form a network structure in the composite material, which improves the compatibility of the interface between the fiber and the matrix, thereby reducing the chance of water molecules entering the interface. For example, researchers discussed the diffusion barrier response of carbon nanotubes in epoxy resin composite materials, and the results showed that the introduction of the carbon nanotubes significantly slowed down the diffusion rate of water molecules and reduced the moisture absorption performance of the composite material [24]. By optimizing the dispersion and content of carbon nanotubes, the diffusion barrier effect can be further improved. Third, the protective layer formed by carbon nanotubes coating on the surface of ramie fibers can also prevent water molecules from entering and reduce the chance of water molecules entering the fibers.

### 3.2. Impact of Moisture Absorption on Interface Strength

**Figure 2:**
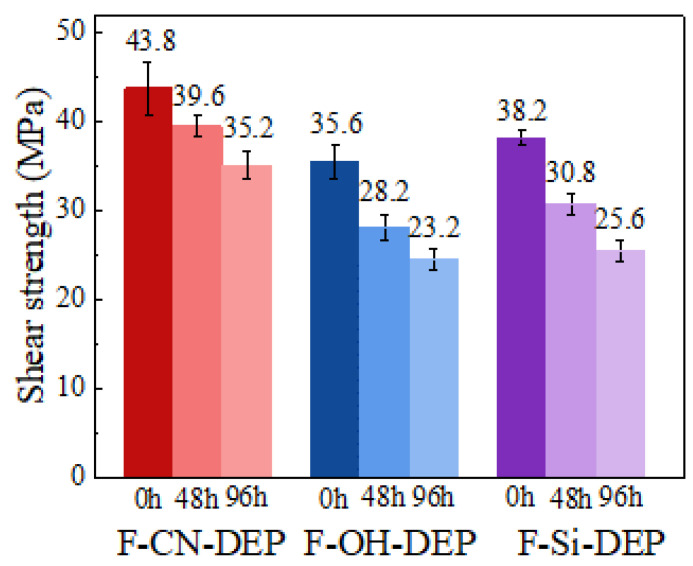
The impact of moisture absorption on the shear strength of different materials. The error bars represent the standard deviations of five representative measurements.

The penetration of water molecules into the material can damage the interface strength between the ramie fiber and the degradable epoxy resin, leading to crack propagation and interface delamination. Therefore, moisture absorption reduces the shear strength of the material and may even result in the interface failure of the composite material. This is one of the urgent issues that need to be addressed in natural fiber/degradable epoxy composite materials. As shown in Figure 2, when the specimens were exposed to a humid environment (temperature of 70 °C, humidity of 80%), the shear strength of the three samples significantly decreased over time. Among them, the short-beam shear strength of the F-CN-DEP decreased from the original 43.8 MPa to 35.2 MPa. This decrease is significantly lower than that of the F-OH-DEP (which decreased from 35.6 MPa to 23.2 MPa) and the F-Si-DEP (which decreased from 38.2 MPa to 25.6 MPa).

**Figure 3:**
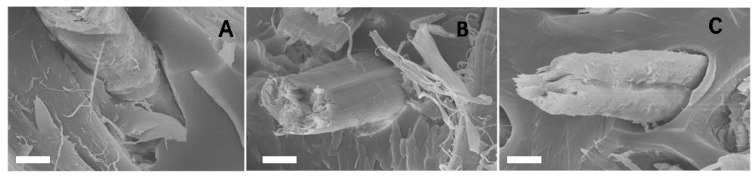
Morphology of fibers in the composite material: (**A**) F-CN-DEP, (**B**) F-OH-DEP, and (**C**) F-Si-DEP. All the scale bars are 5 μm.

The shear strength of a composite material depends on the bonding strength between the fiber and the resin interface. When the bonding strength between the fiber and the resin interface is low, there may be weak bonding or inadequate bonding, resulting in gaps or slippage between the fiber and the resin, leading to weak macroscopic shear resistance. As seen in Figure 3, the fiber surfaces of F-CN-DEP and F-Si-DEP are relatively rough, which is more conducive to the bonding between the fiber and the resin matrix. Compared to the F-CN-DEP, some voids existed in the interface between the silane coupling agent-modified fiber and degradable epoxy, which would have had a bad effect on the interface strength and moisture resistance of the composite material. Combining the results shown in Section 3.1, this experiment demonstrated that carbon nanotube modification enhanced the interface strength and moisture resistance of the composite material. Furthermore, it was further demonstrated that dispersing hydroxylated carbon nanotubes on the surface of ramie fibers can form a barrier layer on the fiber surface or the composite material surface, reducing the penetration of water molecules and reducing the risk of interface delamination and slippage under shear loading.

After the composite material absorbs water, the absorbed water evaporates through thermal action. Under high-temperature conditions, the evaporation of the water causes pressure changes inside the material, leading to expansion and accumulation of stress, resulting in the fracture and failure of the composite material. The improvement of interface strength and the formation of barrier membranes can improve the resistance of the resin matrix and the interface to the swelling effect caused by resin water absorption, thereby enhancing the dimensional stability of the composite material. Table 2 shows the dimensional change rates in the fiber direction (X direction) and the thickness direction (Y direction) of the specimens after being exposed to a humid environment. Among them, F-CN-DEP exhibits the lowest dimensional change in the Y direction, which is consistent with the change in the shear strength of the composite materials.

### 3.3. Influence of Surface Treatment on Heat Resistance

**Figure 4:**
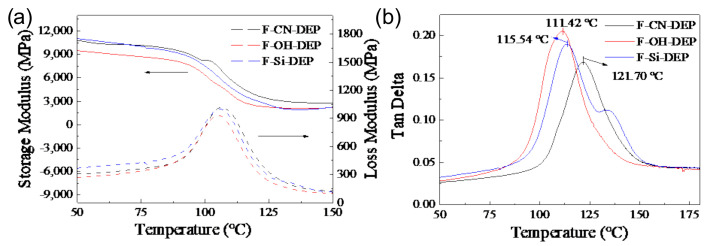
Storage and loss modulus curves (**a**) and DMA thermograms (**b**) for tan delta, against temperature, of different materials.

Figure 4a demonstrates that the storage moduli and loss moduli of the samples are ranked from high to low at the measured temperature ranges as follows: F-CN-DEP > F-Si-DEP > F-OH-DEP. The DMA results indicate that ramie fiber/degradable epoxy composite materials modified with carbon nanotubes exhibit higher heat resistance. The strength of the composite material interface mainly depends on the bonding mechanism and chemical compatibility of the interface. When the interface bonding strength is high, the bond between the fiber and the resin is stronger, allowing for the effective transfer of external loads and an increase in the storage modulus of the composite material [25]. Similarly, when the interface bonding area is larger, there are more bonding points between the fiber and the resin, resulting in a stronger interface bond and better transfer of external loads, thereby increasing the storage modulus of the composite material [26]. The dispersion of carbon nanotubes on the surface of ramie fibers enhances the bonding strength between the fiber and the resin matrix, and its high specific surface area increases the bonding area, thereby improving the storage modulus of the material. Related studies have shown that when the bonding ability at the fiber–resin interface is good, the shear stress transfer at the interface is significantly improved [27,28,29]. The frictional force between the fiber and the resin at the interface increases, allowing for better energy transfer from the fiber to the resin, thereby increasing the loss modulus of the material. Additionally, when the bonding ability at the interface is good, interface failure behavior is effectively suppressed, which is beneficial for improving the loss modulus of the material. In addition, the peak temperature of tan delta (storage modulus/loss modulus) against the temperature curves is defined as the glass transition temperature (*T*_g_). Figure 4b shows that the *T*_g_ of the F-CN-DEP is 121.70 °C, higher than the *T*_g_ values of the F-Si-DEP, at 115.54 °C, and the F-OH-DEP, at 114.42 °C, which further demonstrates that the introduction of carbon nanotubes enhances the binding strength between fibers and the resin matrix.

**Figure 5:**
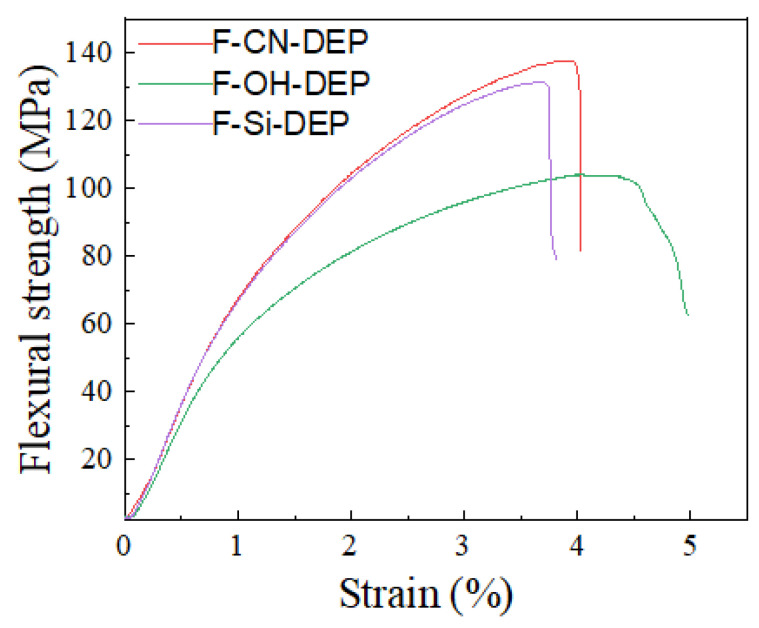
Stress–strain curves of different composites.

The test results of the flexural strengths of the composite materials after the fiber surfaces were treated with different processes are shown in Figure 5. It can be seen from this figure that the elastic moduli of the F-CN-DEP and the F-Si-DEP are 7.5 GPa and 6.9 GPa, respectively; they are relatively stable as the strain increases; and the overall performance is brittle. In comparison, the elastic modulus of F-OH-DEP decreases relatively obviously with the increase in strain and the strength decreases. This is because the fiber and resin in F-CN-DEP and F-Si-DEP are strongly bonded and the interface can effectively transmit the load during the bending deformation process, improving the strength and elastic modulus of the composite material.

### 3.4. Thermal Stability

**Figure 6:**
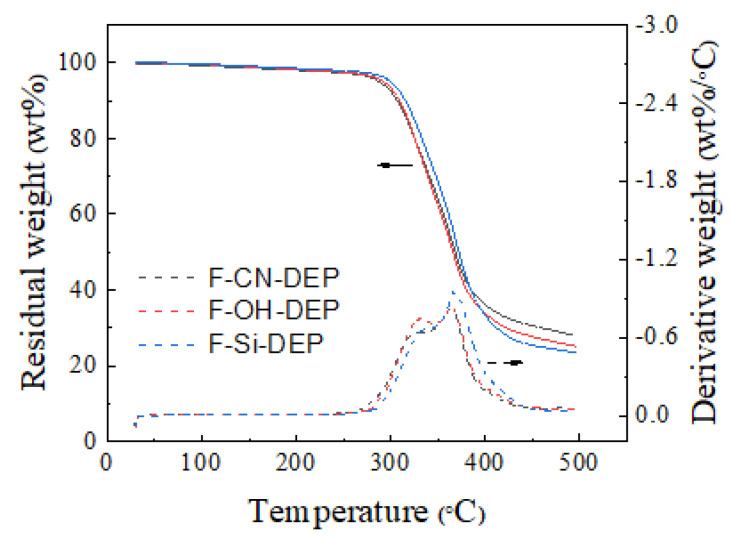
TGA curves of different materials.

Thermal stability is an important indicator for evaluating the heat resistance of materials, determining the maximum temperature they can withstand during use. Figure 5 shows the thermogravimetric analysis curves of different composites. It can be observed that all three composites display two-step decomposition stages. The first stage of decomposition occurs at temperatures around 30−150 °C, which is associated with the evaporation of moisture from the composites. Subsequently, the second stage of decomposition, in the range of 150−400 °C, is attributed to the decomposition of the major constituents of the natural fibers. From Figure 6, it is evident that there is negligible variance in the thermal degradation across the three composites. In the DTG curve, the thermal weight-loss rate curves of the three samples all exhibit similar double peaks, indicating a basically consistent degradation mechanism. Table 3 summarizes the key parameters such as the initial decomposition temperature (*T*_d5_), the temperature at 30% decomposition (*T*_d30_), the temperature at the maximum decomposition rate (*T*_max_), and the residue at 450 °C (C_y450_) for the F-CN-DEP, F-OH-DEP, and F-Si-DEP. The difference lies in the fact that the *T*_d5_ and *T*_d30_ of the F-Si-DEP are higher, which is due to the larger bond energy of the silicon–oxygen bond, which requires a substantial amount of energy to break, resulting in a higher initial decomposition temperature and better heat resistance for F-Si-DEP. These results were further demonstrated by surface morphology characterization (Figure S1) after thermal treatment.

### 3.5. Effect of Surface Treatment on Impact Resistance

**Figure 7:**
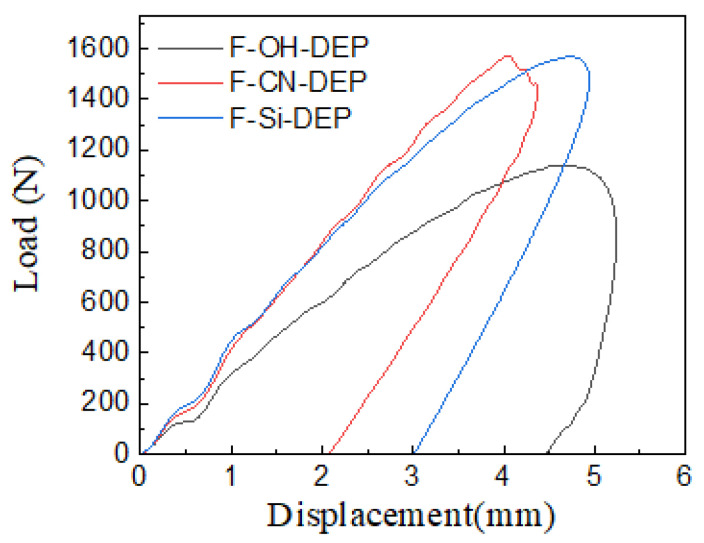
Displacement–load curves during the impact processes of different materials.

Non-penetrating impacts are called low-velocity impacts [30] and may be caused by the impingement of a liquid jet, dropping a tool on a composite structure, or wind gust-stirred debris striking an aeronautic or ground vehicle. Interfacial strength significantly affects the impact resistance of composite materials, and optimizing the interfacial strength can effectively improve the impact resistance of these composite materials [31]. As shown in Figure 7, the F-CN-DEP and the F-Si-DEP exhibit similar initial stage slopes during the first impact process, indicating similar contact stiffnesses. However, the curve of the F-OH-DEP exhibits a lower slope. As for the F-CN-DEP, it reaches the load peak first and the yielding stage duration is short, demonstrating a certain degree of brittleness. But the F-OH-DEP has a longer yielding stage and a lower load peak. This further indicates that F-CN-DEP has a high interfacial strength, making it less susceptible to fiber and resin slippage during impact, while the prolonged yielding stage of F-OH-DEP is mainly due to the ease of fiber and resin slippage, resulting in higher energy consumption during impact.

**Figure 8:**
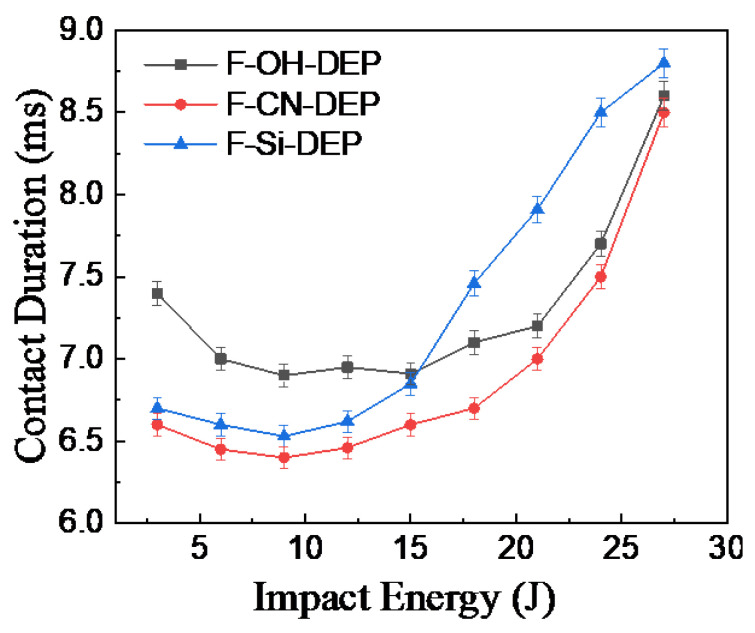
Contact time–impact energy curves of different materials. The error bars represent the standard deviations of five representative measurements.

Furthermore, the contact duration of the F-CN-DEP, F-OH-DEP, and F-Si-DEP during repeated drop impact tests at different energy levels is shown in Figure 8. When the energy is between 3 J and 9 J, the contact duration of the three materials decreases. When the energy is between 9 J and 21 J, the contact duration of these three materials remains consistent. When the impact energy exceeds 21 J, the contact duration rapidly increases with the increasing energy. During the hammer impact, the fibers and resin underwent a series of complex changes. In the initial stage, the impact load was rapidly transmitted to the fibers and resin in the composite material, causing them to experience stress and deformation. At that stage, the fibers bore the majority of the tensile and bending stresses, while the resin bore the shear and compression stresses. As the material entered the energy absorption stage, the deformation of the fibers and resin gradually increased, and the shear and deformation at the interface intensified, allowing the material to begin absorbing energy. When the impact load reached a certain level, the deformation of the fibers and resin reached its limit and they could no longer absorb energy, leading to material failure. When the impact load acted on the composite material, the deformation of the fibers and resin caused relative displacement between them, resulting in interfacial shear. Interfacial shear leads to stress concentration near the interface, causing interface debonding, the formation of microcracks, and even interface failure [32]. When the energy was less than 9 J, the material primarily consumed impact energy through the deformation of the fibers and resin. Due to the high interfacial adhesion strength between the fibers and resin in the F-CN-DEP, impact energy can be rapidly dispersed, resulting in a shorter contact duration. When the impact energy was between 9 J and 21 J, the material primarily absorbed energy through the deformation of its components and interfacial shear, resulting in a relatively stable contact duration. The strong interfacial strength of F-CN-DEP led to a relatively shorter contact duration. As the impact energy increased, the material underwent failure, leading to a rapid increase in contact duration.

## 4. Conclusions

This article provides a detailed analysis and comparison of three components for the surface treatment of natural fibers, namely hydroxyl-functionalized carbon nanotubes, a KH560 silane coupling agent, and alkali treatment, and their effects on the moisture absorption process, interfacial strength, and thermal resistance of ramie fiber/biodegradable epoxy resin composites. Through morphological analysis and shear strength testing, it was found that the composite material treated with carbon nanotubes (F-CN-DEP) exhibited the lowest moisture absorption rate; the highest shear strength, of 43.8 MPa; a high elastic modulus of 7.5 GPa; and a glass transition temperature (*T*_g_) of 121.7 °C. Therefore, it can be concluded that the surface modification method using hydroxyl-functionalized carbon nanotubes can significantly improve the wet-heat stability and mechanical properties of ramie fiber/biodegradable epoxy resin and is advantageous in enhancing contact stiffness and resistance to low-velocity impact during the impact process.

## Figures and Tables

**Table 1:** Abbreviations of composite materials obtained by different processing methods.

Treatment Methods	Abbreviations of Different Composites
Hydroxylated carbon nanotubes	F-CN-DEP
Sodium hydroxide	F-OH-DEP
Silane coupling agent	F-Si-DEP

**Table 2:** Dimensional changes in different composites after moisture absorption.

Sample	Dimensional Change Rate in X (%)	Dimensional Change Rate in Y (%)
F-CN-DEP	0.027	0.581
F-OH-DEP	−0.029	0.863
F-Si-DEP	0.052	0.935

**Table 3:** Key parameters of different composites, obtained from TGA curves.

Sample	*T*_d5_ (°C)	*T*_d30_ (°C)	*T*_max_ (°C)	*T*_s_ (°C) ^1^	*C*_y450_ (%)
F-CN-DEP	287	340	364	156.2	30.6
F-OH-DEP	292	339	364	156.9	27.7
F-Si-DEP	299	348	368	160.9	25.5

^1^ *T*s = 0.49 [*T*_d5_ + 0.6 × (*T*_d30_ − *T*_d5_).

## Data Availability

Data are contained within the article.

## Supplementary Materials

The following supporting information can be downloaded at: https://www.mdpi.com/article/10.3390/ma17081779/s1. Figure S1. Maximum water content (*Mm*) and diffusivity (*D*) of different composites at room temperature. Table S1. Maximum water content (*M_m_*) and diffusivity (*D*) of different composites at room temperature. Ref. [23] is cited in the supplementary materials.
