# Supplementary material for: Influence of Interface Modification on the Moisture Absorption and Thermal Resistance of Ramie Fiber/Degradable Epoxy Composites
Source: Materials (Basel). 2024 Apr 12;17(8):1779. doi: 10.3390/ma17081779 (PMC11051379; doi:10.3390/ma17081779)
Supplement: Supplementary file 1 [file materials-17-01779-s001.zip › materials-2945139-supplementary.pdf]

Influence of Interface Modification on the Moisture Absorption and Thermal Resistance of Ramie Fiber/Degradable Epoxy Composites

Jingqi Geng and Yingchun Cai*

Key Laboratory of Bio-Based Material Science and Technology, Ministry of Education, School of Material Science and Engineering, Northeast Forestry University, Harbin 150040, China;
*Correspondence: caiyingchun@nefu.edu.cn

Table S1. Maximum water content (M_m) and diffusivity (D) of different composites at room temperature.

Sample	M_m (%)	D (10^{-12} m^2/s)
F-CN-DEP	25.98	21.68
F-OH-DEP	24.36	14.31
F-Si-DEP	15.71	13.48

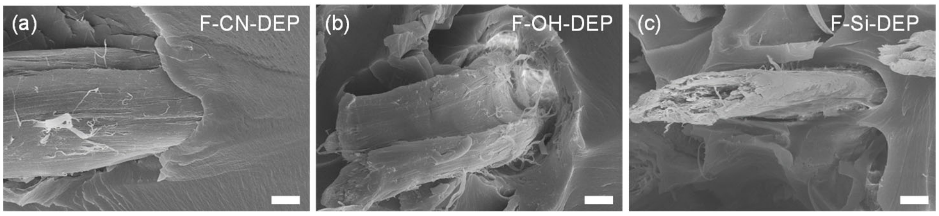

Figure S1. Maximum water content (M_m) and diffusivity (D) of different composites at room temperature.

1

(1) Saikia, D. Studies of water absorption behavior of plant fibers at different temperatures. *International Journal of Thermophysics* **2010,** *31* (4), 1020-1026.
